# Isolation, adaptation, and characterization of lumpy skin disease virus from cattle in Bangladesh

**DOI:** 10.5455/javar.2023.j710

**Published:** 2023-09-30

**Authors:** Sumaiya Pervin, Md. Mostakin Ahamed, Chandra Shaker Chouhan, Md. Salim Jahan, Rony Ahmed, K. H. M. Nazmul, Hussain Nazir, Mahbubul Pratik Siddique, Md. Tanvir Rahman, Md. Abdul Kafi, Md. Bahanur Rahman

**Affiliations:** 1 Department of Microbiology and Hygiene, Faculty of Veterinary Science, Bangladesh Agricultural University, Mymensingh, Bangladesh; 2 Department of Medicine, Faculty of Veterinary Science, Bangladesh Agricultural University, Mymensingh, Bangladesh

**Keywords:** Bangladesh, cattle, lumpy skin disease virus, MDBK cell line, viral attachment protein gene, Vero cell line

## Abstract

**Objective:**

The research aimed to isolate, adapt to cell culture, and characterize the lumpy skin disease virus (LSDV) from clinically infected cattle in Bangladesh.

**Materials and Methods:**

From September 2019 to June 2020, 37 skin nodules and skin swabs were aseptically collected from afflicted cattle in the outbreak regions of Jhenaidah and Kishoreganj in Bangladesh. The LSDV was isolated from embryonated specific pathogen-free (SPF) chicken eggs along the chorioallantoic membrane (CAM) route and the Vero cell line after several blind passages. The viral attachment protein was targeted for molecular detection using polymerase chain reactions (PCR). For phylogenetic analysis, PCR-positive products were partially sequenced.

**Results:**

The virus was evident in the cell line, showed cytopathic effects after the 13 blind passage, and on the CAM of SPF chicken eggs, exhibited thickening of the CAM with pock-like lesions. A total of 12 samples (32.43%) tested positive for LSDV by PCR. Phylogenetic analysis of the present isolates (accession numbers MN792649 and MN792650) revealed 100% similarity with strains from India (MN295064), Kenya (AF325528, MN072619, KX683219), Greece (KY829023), Serbia (KY702007), and Kazakhstan (MN642592); moreover, 99.43% to 100% similarity to the sheep pox virus.

**Conclusion:**

Partially sequenced LSDV was developed as a vaccine seed and was first isolated in Bangladesh and characterized at the molecular level.

## Introduction

Lumpy skin disease (LSD) is a contagious viral infection, transmitted through vectors, capable of crossing international borders, and by the lumpy skin disease virus (LSDV) [1,2]. The original strain of LSDV, the Neethling virus, has just one serotype and is antigenically similar to goat and sheep pox viruses but phylogenetically distinct [2,3]. LSDV has a limited host range and is infectious to all genders and ages of cattle; it does not infect nonruminant hosts [4]. Young animals are more susceptible to severe forms of LSD [5]. This viral disease has hindered the sustainable livestock industry through significant economic losses [3,6]. Because of its tremendous chances for swift spread and consequential financial effects, the World Organization for Animal Health has already registered LSD as a reportable disease [7].

The first outbreaks of LSDV were reported in Zambia, in 1929, and have since been recognized in several African countries [5]. The illness has expanded outside of Africa to a greater extent since 2012, with epidemics in several Middle Eastern countries, including Lebanon, Jordan, and Israel, from 2012 to 2013 [5]. In 2013, it dispersed throughout all of Turkey, where it is now considered an endemic disease [8]. Since 2019, the disease has become prevalent in Asian nations such as India, China, Myanmar, Bangladesh, Nepal, and Pakistan [7,9–13]. LSD first appeared in Bangladesh in September 2019, while the original manifestation can be traced back to July 2019, with the official recognition occurring in August 2019 [11,14].

LSDV is a virus composed of double-stranded DNA with an entire genome of about 151 kilobase pairs (kbp) and 156 probable genes in the central coding region [15]. The central coding region of the LSDV genome, akin to other poxviruses, is surrounded on both ends by two similar homologous repeats in an inverted arrangement, each spanning about 2.4 kbp [15–17].

LSD constitutes one of Bangladesh‘s most economically significant emergent livestock diseases due to its swift and unusual spread [6,11,14,18,19]. In Bangladesh, only a small number of studies on LSD‘s epidemiology, economic effects, pathology, and molecular scrutiny are currently carried out and reported [6,11,14,18,19]. It was also reported that cell cultures, such as Madin-Darby bovine kidney (MDBK) cells, can be used to isolate LSDV, and quantification and titer can be determined [20]. For effective disease control strategies, it is imperative to identify and understand the specific field viral strain(s) prevalent in Bangladesh that are responsible for outbreaks. However, as far as our current understanding goes, there have been no documented reports regarding the isolation, adaptation to culture, and molecular characterization (during the study period) of LSDV obtained from cattle in Bangladesh.

## Material and Methods 

### Ethical approval

All laboratory work was conducted at the “Virology Laboratory, Department of Microbiology and Hygiene, Bangladesh Agricultural University (BAU), Mymensingh-2202, Bangladesh, following the standard procedures. The Institutional Ethical Committee [approval number AWEEC/BAU/2019 (52)] approved using live animals and cell cultures in the laboratory experiment.

### Study period and areas

The research was done in the various regions of Jhenaidah (23.5450°N, 89.1726°E) and Kishoreganj (24.4333°N, 90.7833°E) districts in Bangladesh from September 2019 to March 2020. Regular monitoring and discussion with veterinary professionals at the particular district-level veterinary hospital were used to evaluate outbreaks.

### Sample collection

A total of 37 samples (Jhenaidah: 24 samples; Kishoreganj: 13 samples) of skin biopsies from cutaneous nodules and nodule swabs were taken from cattle with clinical signs suspected of infection with the LSD virus. The sample collection and processing procedures were done per standard procedure [19]. After cleaning nodules and disinfecting the surface areas with a 70% isopropyl alcohol solution, skin biopsies, and sterile cotton specimens were obtained in an aseptic manner using a sterilized scalpel blade from the cutaneous nodules of each representative cattle. Tissue samples were placed in 10 ml of viral transport media in a sterile 15 ml falcon tube containing 5% inactivated fetal bovine serum (FBS) with a cold chain system. After shipment, the viral samples were stored at −80°C until further analysis [20].

### Sample processing and viral inoculum preparation

The obtained samples from skin biopsies were thawed at 25°C and then cleaned in an aseptic manner with PBS (pH 7.2) [21]. A tissue suspension with a concentration of 20% (weight/volume) was prepared by grinding 2 gm of tissue in a sterile mortar and pestle and then mixing it with 8 ml of sterilized PBS while stirring [20]. Supernatants were collected from the processed suspensions following centrifugation at 6,000 rpm for 10 min at 4°C and treated with penicillin and streptomycin at a concentration of 1,000 IU/ml and 1 mg/ml, respectively, for at least 1 h to make samples free from bacterial contamination. Collected supernatants were inoculated in soybean-casein digest broth and incubated at 37°C to check for sterility. Suspensions were divided and kept at −20°C until they were used for virus growth and isolation [20,22].

### Virus isolation and propagation

Chicken egg embryos around 10 days old were used for virus isolation and propagation [23]. The specific pathogen-free (SPF) fertile chicken eggs were collected from Incepta Vaccine Ltd., Bangladesh. The chorioallantoic membrane (CAM) route was used to inoculate the prepared raw samples [22,24]. Therefore, 200 μl of viral inoculums were accounted for for inoculation through the CAM route of the SPF egg. Eggs that had been inoculated underwent incubation at 37°C, and embryo survivability was monitored for 5–6 days after inoculation. Embryos that perished within 24 h of assessment were eliminated, and those that were viable after 24 h to 6 days of incubation were chilled at 4°C overnight [20,22,24]. Only CAMs were gathered and pulverized using a sterile mortar and pestle to prepare a homogenized mixture of CAMs. The tissue samples were centrifuged at 4°C for 10 min at 6,000 rpm. Then, the collected supernatants were aliquoted and kept at −80°C [22,25].

### Virus adaptation in cell lines

Vero and MDBK cell lines were utilized to isolate and adapt viruses in continuous cells via numerous blind passages [20,26]. Incepta Vaccine Ltd. and the Department of Microbiology and Hygiene, BAU, supplied MDBK and Vero cells. The cell line inoculum was formed by centrifuging an unprocessed specimen and extracting the supernatant in a fresh centrifuge tube with 100 gm/ml of the antibiotic (gentamicin). Cell culture‘s confluent growth was achieved by mixing Minimal Essential Media (MEM) with 10% PBS and incubating at 37°C in a CO_2_ (5%) incubator. At confluent cell growth, a 500-µl of virus inoculum was injected into cells supplemented with MEM and 5% FBS and incubated at 37°C with 5% CO_2_ for virus adaptation, and several consecutive passages were carried out until the cell‘s cytopathic effect (CPE) was observed [20,22]. After 48–72 h postinfection (hpi), when the CPE was greater than 75%, the inoculated containers were examined using an inverted microscope (Carl Zeiss, Germany). Multiple freezing and thawing cycles were used to extract viruses stored at −80°C for future use. Consequently, the cytopathic response witnessed through consecutive passages in the cell line was utilized to recognize the infection of the virus through its propagation [20,24].

### DNA extraction and real-time polymerase chain reactions (PCR)

From a 20% tissue suspension of the field samples, infected embryonated eggs, and infected cell culture fluid, a commercially available kit (Wizard^®^ Genomic DNA Purification Kit, Promega, USA) was used for the extraction of the LSD virus DNA following the manufacturer‘s instructions. PCR was done to amplify the target sequence of the viral attachment protein gene in the DNA of LSDV [20]. At the Central Disease Investigation Laboratory in Dhaka, real-time PCR was employed to validate the positive samples. A reaction mixture of 25 µl volume was used for PCR, consisting of 5.5 µl of nuclease-free water, 1 µl of forward primer (5¢-TCC GAG CTC TTT CCT GAT TTT TCT TAC TAT- 3¢), 1 µl reverse primer (5¢-TAT GGT ACC TAA ATT ATA TAC GTA AAT AAC-3¢), 12.5 µl of 2X qPCR Go Taq Master Supermix (Promega, USA), and 5 μl of DNA template [27]. Amplification of DNA was conducted with an initial 10 min of denaturation of the product at 95°C, followed by 45 cycles of the reaction as denaturation for 15 sec at 95°C and annealing of the primer at 60°C for 45 sec [27]. Furthermore, the denaturation process of the product was conducted at 95°C for 1 min (held for 1 min), cooled to 40°C for 1 min, and continuously heated at 0.5°C for 10 sec to acquire fluorescence from 45°C to 85°C. The IAEA Animal Health Laboratory, Vienna, obtained positive internal control for the real-time PCR reaction [25].

### Agarose gel electrophoresis

The PCR-amplified products were scrutinized through agarose gel electrophoresis. 5 µl of amplified PCR outcomes were incorporated with 1 l of 6× loading dye (Promega, USA) and deposited onto an agarose gel well. Ethidium bromide was used to stain DNA in the gel after electrophoresis completion, and a UV-transilluminator (Biometra, Germany) was used to visualize stained DNA [20].

### Sequencing and phylogenetic analysis

PCR products were commercially sequenced for molecular characterization and uploaded to GenBank. Sequenced data were assembled and aligned using Codon Code Aligner and Nucleotide basic local alignment search tools. Using the neighbor-joining approach, phylogenetic analysis was done using MEGA X software against related GenBank sequences [28,29]. The bootstrap consensus tree constructed from 1,000 replicates represents the assumption of the evolutionary history of the texa [30]. Evolutionary distances were determined utilizing the *p*-distance technique and are represented as base differences per site unit [28,29]. All gaps and absent data were eliminated (with the option for complete deletion).

## Results

### Identification of viruses

**Figure 1.**
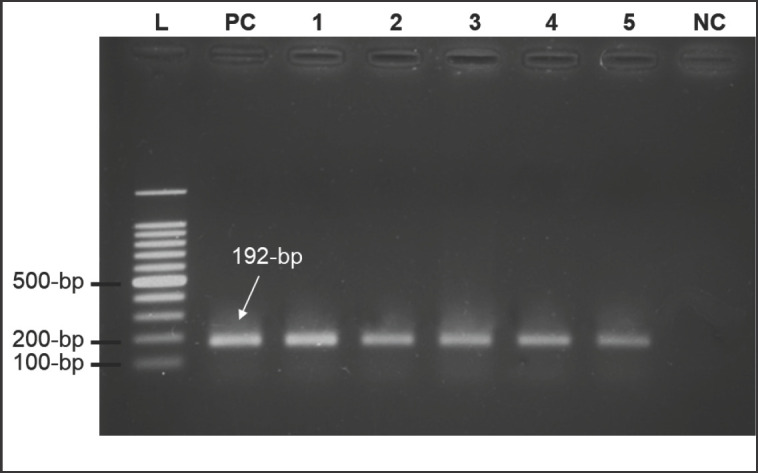
PCR products of the attachment gene (192-bp) of five LSDV isolates (lane 1 to 5) in ethidium bromide stained agarose gel (1.5% *w*/*v*) electrophoresis, and 100-bp DNA ladder (lane L), positive control (lane PC), and negative control (lane NC).

Field isolates and re-isolated viruses from adapted cell lines and SPF eggs were used for molecular characterization using their extracted DNA with specific primers that showed bands at the 192-bp region after gel electrophoresis. Real-time PCR was performed to verify the positive outcomes (Figure 1). The virus‘s identity was determined as LSDV in light of its characteristics, clinical signs, and LSDV-specific genomes.

### Virus isolation

**Figure 2.**
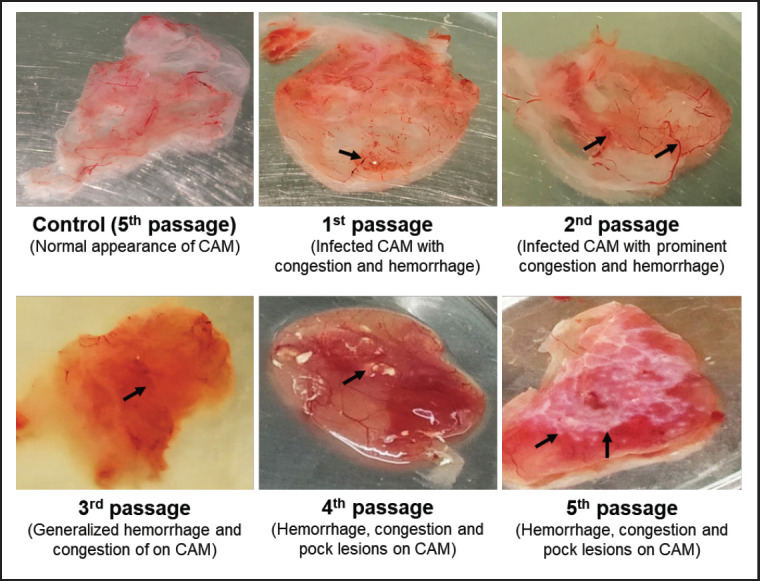
LSDV-infected CAM showing hemorrhage with pock lesions at different passage, and control CAM showing no hemorrhage.

After 3 to 5 days following inoculation, the embryonated eggs die. The CAM was expanded by hemorrhagic lesions encompassing the whole membrane and uneven congestion. The LSDV lesion on CAM ranged from membrane thickening in the first passage to multiple white foci, more evident in the second and third passages (Figure 2). Besides, live embryos were found in uninfected control eggs. The CAM showed no signs of hemorrhage, and the PCR results were negative. Twelve cattle (32.43%) screened positive for the virus, which was propagated in SPF eggs and cell culture and then confirmed by PCR and quantitative real-time polymerase chain reaction using a specific primer. 9 out of 24 cattle (37.5%) tested positive for the LSDV virus in Jhenidah, and 3 out of 13 (23.707%) tested positive in Kishoreganj.

### Adaptation to Vero and MDBK cell lines

**Figure 3.**
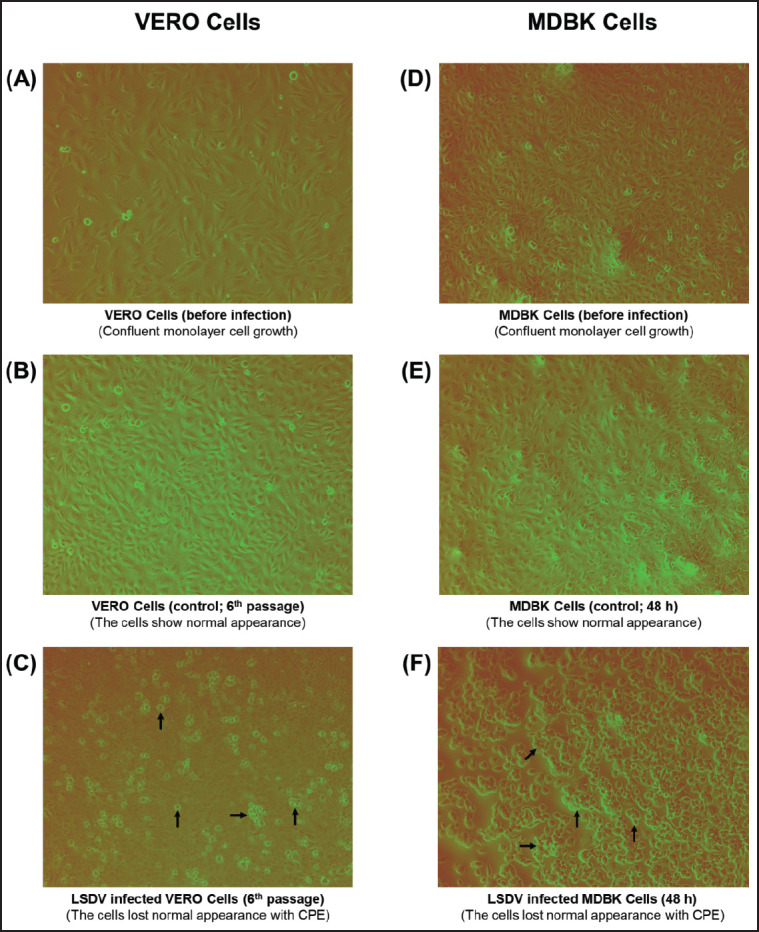
Cell culure adaptation of LSDV. (A) Normal Vero cells showing confluent growth before infection, (B) LSDV Control Vero cells after 72 h of infection, (C) LSDV-infected Vero cells after 72 h of infection, (D) Normal MDBK cells showing confluent growth before infection. (E) LSDV Control MDBK cell after 48 h of infection, (F) LSDV-infected MDBK cell after 48 h of infection.

To produce the desired titer of the virus, an adaptation of the virus should be carried out in established cell lines to overcome the inadequacy of SPF eggs and the short-term survival of primary cell lines. LSDV was developed by inoculating MDBK and Vero cell lines with naturally infected cattle samples. The Vero cell demonstrated a characteristic CPE within 72 h of infection from 5 to 6 passages (Figure 3). Another highly recommended MDBK cell showed CPE within 48 h after inoculation from the initial passage. The infected cells became round and aggregated, which defined the CPE, which appeared as clusters that spread over the monolayer and subsequently enlarged (Figure 3). In contrast, the cells in the control flask were found to be adhesive to the surface of the cell culture flask.

### Phylogenetic analysis

**Figure 4.**
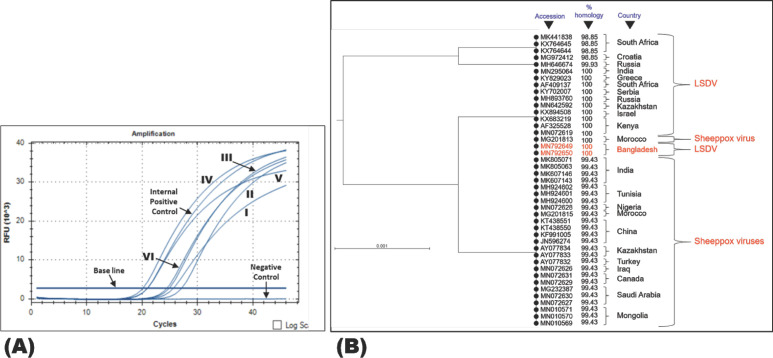
Cycle threshold for the samples were 25.61 for I, 27.13 for II, 24.43 for III, 20.14 for IV, 20.97 for V, 24.89 for VI, and 20.96 for positive control (A). Phylogenetic analysis of LSDV (B).

The commercially sequenced PCR products were analyzed, assembled, and uploaded to GenBank (accession numbers MN792649 and MN792650). Phylogenetic analysis of LSDV showed 100% similarity with MN295064 (India); AF325528, MN072619, and KX683219 (Kenya); KY829023 (Greece); AF409137 (South Africa); KY702007 (Serbia); KX894508 (Israel); MN642592 (Kazakhstan); and 98.85%–99.93% similarity with the sequence data published from South Africa (MK441838, KX764644, KX764644, and KX764645), Croatia (MG972412), and Russia (MH646674) (Figure 4). In addition, the LSDV of this study demonstrated 99.43% to 100% similarity to the sheep pox virus (Figure 4), demonstrating that LSDV and sheep poxviruses are genetically similar.

## Discussion

The LSDV pandemic is inextricably entwined with food safety and cattle rearing because it threatens food security and causes financial losses to the livestock sector [6]. This study isolated LSDV in cattle by virus culture in SPF eggs and continuous cell lines, namely Vero and MDBK, for the first time in Bangladesh.

For LSD virus isolation, SPF egg (Thailand; supplied by Incepta Vaccine Ltd., Bangladesh) and two continuous cell lines, such as Vero and MDBK (supplied by Incepta Vaccine Ltd., Bangladesh), were used for the first time in Bangladesh. Except for primary cell lamb testis described in Bangladesh, no available data about LSD virus isolation in SPF eggs or cell lines was found during this study [25]. The primary cell was not used in this study for isolation because it is not generally used for several passages as a continuous cell, and it is an expensive process for preparation. Instead, SPF eggs (Thailand) and continuous cell lines such as Vero and MDBK were used. Moreover, the primary cell tends to get contaminated easily. Our study aimed to produce a desired virus titer in a recommended cell line as described for vaccine development from local isolates [20,26,31,32]. As for propagation in SPF eggs, it took 3–5 passages for adaptation, and there were characteristics of pock lesions on CAM, as described by Ateya et al. [32]. For virus isolation and culture, the LSDV samples were inoculated into 10-day-old SPF embryonated chicken eggs (Thai SPF, Thailand) via the CAM route [27,32]. In addition, the characteristic pock lesion and changes (e.g., hemorrhage and thickening) were observed 6 days postinoculation at the fourth passage, as reported previously [23,32].

CPE, as described by Kumar et al. [20], was observed, as was CPE in another highly recommended MDBK cell, within 72 h after inoculation from the first passage, as described in the previous report [20,32]. The CPE was distinguished through cell rounding and aggregation, which consolidated into clusters dispersed throughout the monolayer and extended progressively. In contrast with the cells of control flasks, which were found to be live and adherent to the flask surface, the LSDV-adapted cells showed typical changes (death of 70%–80% of cells and detached cells from the flask surface) under an inverted microscope at 72 h postinoculation. The cells showed characteristic clumping and rounding instead of their typical spindle-like shapes [20,32].

In the phylogenetic analysis, it was observed that the present isolates (MN792649 and MN792650) assembled with 100% similarity with the isolates from India, Kenya [33,34], Greece [35], South Africa [36], and Serbia [37]. Moreover, sequence similarity with previously published sheeppox virus sequences revealed 99.43% homology in most cases, though 100% similarity was found with only one Moroccan sheeppox virus isolate (MG205810), which could be due to their antigenic similarity [2]. In fact, partial sequencing does not provide details or exact genetic differences [12,18], hence the need to explore whole genome sequencing for the present isolates in the future.

## Conclusion

LSD is a problem in many countries around the world. This study identified LSDV in cattle through clinical signs and isolated it by virus culture in SPF eggs and continuous cell lines, namely Vero and MDBK, for the first time in Bangladesh. These local isolates could be vaccine candidates for developing an effective LSD vaccine in Bangladesh. The low-level genetic variability could indicate cross-border transmission of the virus and the sustainability of its virulence.

## List of Abbreviations

BAU, Bangladesh Agricultural University; CAM, chorioallantoic membrane; FBS, fetal bovine serum; LSDV, Lumpy skin disease virus; MDBK, Madin-Darby bovine kidney; MEM, Minimal essential media SPF, Specific pathogen free.
